# Similarity of Center of Pressure Progression during Walking and Jogging of Anterior Cruciate Ligament Deficient Patients

**DOI:** 10.1371/journal.pone.0169421

**Published:** 2017-01-10

**Authors:** Hongshi Huang, Jianwei Qiu, Tianlin Liu, Yuanyuan Yu, Qinwei Guo, Dingsheng Luo, Yingfang Ao

**Affiliations:** 1 Institute of Sports Medicine, Peking University Third Hospital, Beijing, People’s Republic of China; 2 Key Laboratory of Machine Perception (Ministry of Education), Speech and Hearing Research Center, and Department of Machine Intelligence, School of Electronic Engineering and Computer Science, Peking University, Beijing, People’s Republic of China; Mayo Clinic Minnesota, UNITED STATES

## Abstract

**Objective:**

To evaluate the center of pressure (COP) progression similarity and its change during walking and jogging in Anterior Cruciate Ligament deficient (ACLD) patients.

**Methods:**

A study was performed in 64 unilateral ACLD subjects and 32 healthy volunteers who walked and jogged on footscan® system at a self-selected speed. COP trajectory during walking and jogging was calculated. The robustness and similarity scores of COP (SSCOP, similarity scores with respect to corresponding COP trajectories) were computed, and then the Analysis of Variance test was employed to compare among different conditions (left or right side, within a subject or between subjects, walking or jogging).

**Results:**

(1) During the same motion status (walking or jogging), SSCOP were higher than 0.885. However, SSCOP between walking and jogging were lower than 0.25 in both the healthy and ACLD group. SSCOP between the intrasubjects were statistically higher than those between the intersubjects (p<0.01). (2) SSCOP in the ACLD group were statistically significantly reduced to 0.885±0.074 compared to 0.912±0.057 in healthy volunteers during walking, and 0.903±0.066 in the ACLD group compared to 0.919±0.050 in the healthy group during jogging (p<0.01).

**Conclusions:**

SSCOP can distinguish walking from jogging, and SSCOP of ACLD patients would be different from that of healthy controls.

The study protocol was approved by the Institutional Research Board of Peking University Third Hospital (IRB00006761-2012010).

## Introduction

Anterior cruciate ligament (ACL) deficiency is a common sports injury that increases tibiofemoral laxity and leads to knee joint instability, which can affect the performance of daily activities. Several biomechanical studies [1,2,3,4] reported an increased internal rotation of the tibia of the ACL deficient leg during gait. Because of an automatic coupling between tibial internal/external rotation, calcaneus eversion/inversion, and forefoot pronation / supination during stance phase [5,6], ACL deficiency could lead to a change of plantar pressure during gait [7,8].

Gait cycle of the walking and running consists of a stance phase and a swing phase. The percentage of the stance phase varies depending on gait speed—about 60% with walking, 40% with running, and 22% with world class sprinters. The distinction of walking is that gait cycle involves a period of double limb support in which both feet are on the ground. However, there is a period of double float in which both feet are off the ground during jogging or running [9]. The center of pressure (COP) is the point of location of the vertical ground reaction force vector. The COP progression is a trajectory formed by a series of coordinates in the center of pressure as it passes from heel to toe. The position of the center of pressure during the stance phase of gait characterizes the foot progression on the ground. Hence, spatial–temporal characteristics of the COP path may be used to identify balance control, lower limb function, and treatment efficacy [10]. The efficacy of using both plantar pressure devices and force plates to record COP had been demonstrated [11,10,12]. A new COP calculation strategy based on the plantar pressure data was demonstrated to be simpler, saving time and expensive costs, effective and promising[13,14]. In addition, COP trajectories similarities between walking and jogging are very low which indicates COP trajectories can distinguish walking from jogging in healthy volunteers [14]. While numerous studies have outlined biomechanical features in ACL deficiency, the COP dynamic behavior in response to ACL deficiency has been relatively neglected. This investigation attempts to clarify the COP trajectory characteristics for ACL deficiency during barefoot walking and jogging. The effect of unilateral ACL deficiency on the COP progression will be discussed through comparison with uninjured adults using the similarity of the COP progression.

The aim of this study was to describe and quantify the similarity of the COP displacement during walking and jogging in healthy volunteers and in unilateral ACL deficient patients. The similarity of the COP displacements during walking was further investigated whether differed from that during jogging, and if COP similarity in the unilateral ACL-deficient patients was different from that of healthy volunteers.

## Materials and Methods

### Subjects

Thirty-two healthy college students (age: 22.4±0.7 years; body weight = 53.4±8.5 kg) and 64 symptomatically unilateral (33 left and 31 right side) ACL deficient subjects (age: 30.3 ± 9.5 years; body weight = 76.30 ± 14.19 kg) participated in the study. The injured knee was verified with a physical examination by an orthopedic surgeon and with an MRI examination. Additionally, the status of each injured ACL was confirmed during an arthroscopy performed after the completion of this study. All ACL-deficient subjects included in this study had unilateral ACL rupture, accompanied with minimal other tissue injuries that do not need surgical intervention. There was no history of injury, surgery or disease in the contralateral knees. Regular intervention following ACL injury was done. Controlling the knee hemarthrosis and general inflammatory process was the first goal. The patients were given crutches and instructed in a pain-free partial weight-bearing gait. Motion exercises were started with concentration on passive extension to help prevent rapid scarring in the intercondylar notch. Weight bearing were increased as pain and joint effusion decreases, quadriceps control increases, and full active knee extension was achieved. The study protocol was approved by the Institutional Research Board of Peking University Third Hospital (IRB00006761-2012010). After explaining the aims and risks of the study, subjects gave written informed consent. During the test, all patients were free of knee joint swelling and were able to walk and jog without any subjective discomfort. However, they reported symptoms of giving-way during turning or twisting activities.

### Procedures and instrumentation

The subjects walked or jogged barefoot on a two meter footscan® system (RSscan International, 2×0.4 m, 16384 sensors, sample frequency 126 Hz for walking, and 500Hz for jogging), that was placed halfway on a 16.5 meter walkway. Before data collection, subjects warmed up and were asked to perform practice trials until they had familiarized themselves with barefoot walking and jogging. Subjects were asked to walk and jog at a constant (self chosen) speed.

A trial was considered valid when the following criteria were met: (1) at least two complete footprints, (2) presence of a heel-strike pattern, (3) no visible adjustment in gait pattern when crossing the plate [15], (4) total stance duration was within 10% of the individual average value. A total of five valid trials per leg were used for data analysis.

### Data analysis

1) COP Trajectory Generation

The coordinates of COP, noted as *COP*_*X*_ and *COP*_*Y*_, were the anterior-posterior, medio-lateral position of COP, respectively. Theoretically, they could be expressed according to following formula:

$${COP}_{X}=\frac{\iint xF(x,y)\;dxdy}{\iint F(x,y)\;dxdy},\;{COP}_{Y}=\frac{\iint yF(x,y)\;dxdy}{\iint F(x,y)\;dxdy}$$ (1)

where *F*(*x*, *y*) was the continuous pressure distribution in the supporting surface area. While in practice, pressure distribution was detected by pressure-sensor array, which just was a discrete case. To let the calculation of *COP*_*X*_ and *COP*_*Y*_ be possible, formula (1) was then modified as following:

$${COP}_{X}=\frac{\sum_{i,j}{x_{i}F}_{i,j}}{\sum_{i,j}F_{i,j}},\;{COP}_{Y}=\frac{\sum_{i,j}{y_{j}F}_{i,j}}{\sum_{i,j}F_{i,j}}$$ (2)

where *F*_*i*,*j*_ was the discrete pressure distribution in the supporting surface area. Since the discretization may lead to information loss and then debase the accuracy of the calculation, in this research, a new strategy was adopted to calculate the coordinates of COP, as shown in formula (3):

$${COP}_{\left(X,Y\right)}=(x,y)\;|\;min\sum_{i,j}F_{\left(i,j\right)}D((i,j),(x,y))$$ (3)

where both *COP*_*X*_ and *COP*_*Y*_ were denoted as *COP*_(*X*,*Y*)_, whose value will be the (*x*, *y*) that satisfied the minimized process as characterized by the right side constraint of the formula. The vector (*i*, *j*) takes over all the activated sensors, and *D*((*i*,*j*), (*x*,*y*)) was the Euclidean distance between (*i*, *j*) and (*x*, *y*). The origin (0, 0) of the coordinate system was set at the center of the heel.

**Fig 1:**
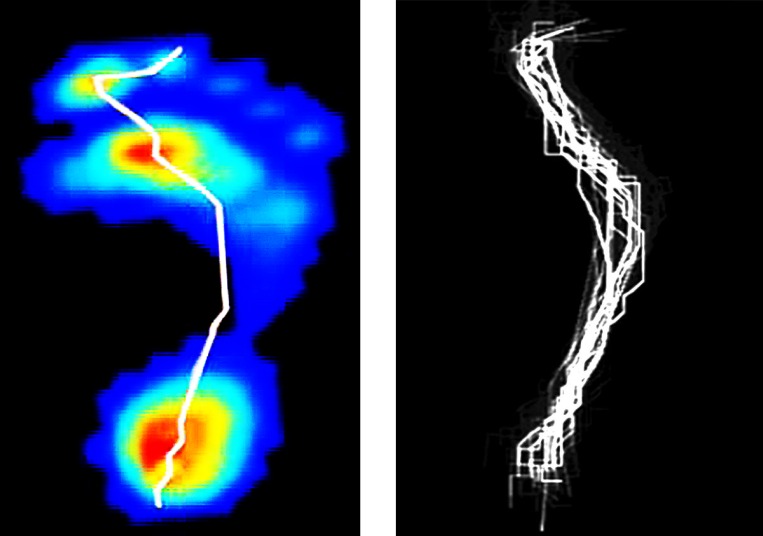
(a, left) COP trajectory of a right foot during walking (b, right) different COP trajectories normalized in unit scale of a right foot during walking.

According to the above mentioned method, the COP point of a specific time frame could be calculated. After all COP points in a trial were calculated, a COP trajectory was then generated (in Fig 1).

2) Similarity Computation

COP trajectories were calculated separately beneath the foot of the affected side or the intact side of patients, and then similarity between two COP trajectories was computed. Similarity between two COP trajectories was defined in formula (4):

$$Sim(S_{1},S_{2})=\frac{\sum_{t}S_{1}(t)∙S_{2}(t)}{\sqrt{\sum_{t}S_{1}^{2}(t)}∙\sqrt{\sum_{t}S_{2}^{2}(t)}}$$ (4)

where *S*_1_ and *S*_2_ were two COP trajectories. Based on this definition, several statistics were defined to observe different aspects of human COP trajectory for a natural walking or jogging behavior. First, to observe the robustness of each person, an individual’s similarity referring to intra-participant similarity was defined as in formula (5):

$${Sim}^{Ind}=\frac{1}{{∁}_{m}^{2}}\sum_{i<j}Sim(S_{i},S_{j}),\;1\leq i<j\leq m,$$ (5)

where *m* was the number of trials taken by each participant (equals to 5 in this research) and *S*_*i*_, S_*j*_ were two intra-participant COP trajectories. To observe the characteristics of COP trajectories of a specific group of participants, the average among all individual similarities within the group was taken according to formula (6):

$${Sim}^{Ave}=\frac{1}{n}\sum_{k=1}^{n}{Sim}_{k}^{Ind},$$ (6)

where *n* was the participants number of the group to be investigated and index *k* refers to the *k*th individual in this group. Alternatively, by averaging similarities of all pair trials of all participants in a given group, a more robust observation of characteristics of COP trajectories of such a group could be achieved by using formula (7):

$${Sim}^{All}=\frac{1}{{∁}_{n\times m}^{2}}\sum_{i<j}Sim(S_{i}^{\prime },S_{j}^{\prime }),\;1\leq i<j\leq n\times m$$ (7)

where $S_{i}^{\prime }$ and $S_{j}^{\prime }$ were two COP trajectories from all trials involving all different participants in a given group.

Similarity scores of COP trajectories of different groups to be investigated were compared under a variety of conditions, which was between different sides (left or right; injured or intact), between intrasubject (within the same subject) or intersubjects (between different subjects), and between walking or jogging.

In order to evaluate whether the COP similarity under Walking motion pattern (marked in “W”) was different from that of Jogging motion pattern (marked in “J”) in Experiment I: On Different Motion Patterns, several groups corresponding to two motion patterns were setup under four conditions: Left side (marked in “L”), Right side (marked in “R”), Healthy volunteers (marked in “H”) and ACL-ruptured patients (marked in “A”). Thus, there was a total of four groups to investigate the difference between two motion patterns, noted as “HL-WJ”, “HR-WJ”, “AL-WJ” and “AR-WJ” respecitvely, where for example, the group labelled with “HL-WJ” referred that the observation were focused on Left side of Healthy subjects by calculating the similarity of COP trajectories between Walking and Jogging for a given subject. As a consequence, four similarity scores according to formula (6) were obtained to characterize four studied groups. Analogously, there was a total of eight groups under same motion patterns (four for each pattern) to be investigated as a comparison, noted as “HL-W”, “HR-W”, “HL-J”, “HR-J”, “AL-W”, “AR-W”, “AL-J” and “AR-J”. These group labels shared the same meanings as abovementioned, for example, the group labelled with “HL-W” referred to that the observation focused on Left side of Healthy subjects during Walking. And then eight similarity scores were calculated according to formula (6) to characterize these eight control groups.

In order to evaluate whether the COP similarity on the ipsilateral side (marked in “I”) was different from that on the contralateral sides (marked in “C”) in Experiment II: On ipsilateral and contralateral, similarity scores were calculated between the same sides and different sides (left or right; injured or intact) of the same subject during walking (marked in “W”) or jogging (marked in “J”). Several groups corresponding to two motion patterns were setup under four conditions: Left side (marked in “L”), Right side (marked in “R”), Healthy volunteers (marked in “H”), and ACL-ruptured patients (marked in “A”). Thus, there was a total of four groups were formed to investigate the difference between the contralateral sides, respectively noted as “HW-C”, “HJ-C”, “AW-C”, and “AJ-C”, where for example, the group labelled with “HJ-C” referred to the observation that focused on the Contralateral sides of Healthy subjects by calculating the similarity of COP trajectories during Jogging. As a consequence, four similarity scores according to formula (6) were obtained to characterize these four studied groups. Analogously, there was a total of eight groups under same sides (four for each motion pattern) to be investigated as a comparison, noted as “HW-IL”, “HW-IR”, “HJ-IL”, “HJ-IR”, “AW-IL”, “AW-IR”, “AJ-IL” and “AJ-IR”. These group labels shared similar meanings as abovementioned, for example, the group labelled with “HW-IL” referred to the observation focused on the Ipsilateral Left side of Healthy subjects during Walking. Eight similarity scores were then calculated according to formula (6) to characterize these eight control groups.

In order to evaluate whether the COP similarity on the sides of intrasubjects (marked in “Intra”) was different from that of the intersubjects (marked in “Inter”) in Experiment III: On intrasubject and intersubject, similarity scores were calculated between different sides of the same subject and those between different subjects during walking (marked in “W”) or jogging (marked in “J”). Several groups corresponding to two motion patterns were setup under four conditions: Left side (marked in “L”), Right side (marked in “R”), Healthy volunteers (marked in “H”), and ACL-ruptured patients (marked in “A”). Thus, there were a total of eight groups formed to investigate the difference between the sides of different subjects, respectively noted as “HWL-Inter”, “HWR-Inter”, “HJL-Inter”, “HJR-Inter”, “AWL-Inter”, “AWR-Inter”, “AJL-Inter” and “AJR-Inter”. For example, the group labelled with “HJR-Inter” referred to the observation focused on the Right side of the Healthy Intersubjects by calculating the similarity of COP trajectories during Jogging. As a consequence, eight similarity scores according to formula (7) were obtained to characterize these eight studied groups. Analogously, eight groups under the same side of the same subject (four for each motion pattern) to be investigated as a comparison, noted as “HWL-Intra”, “HWR-Intra”, “HJL-Intra”, “HJR-Intra”, “AWL-Intra”, “AWR-Intra”, “AJL-Intra” and “AJR-Intra”. These group labels shared the same meanings as abovementioned, for example, the group labelled with “HWL-Intra” referred to the observation focused on the Left side of the same Healthy subjects during Walking. Eight similarity scores were calculated according to formula (6) to characterize these eight control groups.

In order to investigate whether the COP similarity changed following ACL rupture, differences between normal and pathology subjects were studied in Experiment IV: The influences derived from ACL deficiency. Firstly, similarity scores were calculated according to formula (7) for ACL-ruptured patients (marked in “A”) under four conditions: Walking (marked in “W”), Jogging (marked in “J”), Left side (marked in “L”), and Right side (marked in “R”). Thus, four observed groups were formed to investigate the influences derived from ACL-ruptured patients, respectively noted as “WL-inter-A”, “WR-inter-A”, “JL-inter-A” and “JR-inter-A”. For example, the group labelled with “JR-inter-A” referred to the observation focused on the Right side of the ACL-ruptured patients by calculating the similarity of COP trajectories during Jogging. Meanwhile, there were another four groups formed to further investigate such differences by calculating similarity scores between Healthy volunteers and ACL-ruptured patients, respectively noted as “WL-HA”, “WR-HA”, “JL-HA” and “JR-HA”. For example, the group labelled with “JR-HA” referred to the observation focused on the Right side between Healthy volunteers and ACL-ruptured patients by calculating the similarity scores of COP trajectories during Jogging. As a consequence, totally eight similarity scores according to formula (7) were obtained to characterize these eight studied groups. Analogously, there were four control groups under the same sides (two for each motion pattern) of the Healthy subjects to be investigated as a comparison, noted as “WL-inter-H”, “WR-inter-H”, “JL-inter-H” and “JR-inter-H”. These group labels shared the same meanings as abovementioned, for example, the group labelled with “WL-inter-H” referred to the observation focused on the Left side of the Healthy subjects during Walking. Four similarity scores were calculated according to formula (7) to characterize these four control groups.

To be clearly, all results from above experiments were re-exhibited in a new way, where similarity score of a healthy volunteers group under given observation condition was taken as a control group, while that of a ACL deficiency group under the same condition was taken as a studied group. Each group under some observation condition was labeled by same characters just as that used in experiments mentioned above, only with some slight position adjustment to let it lay out more clearly. Moreover, for all observation conditions that involve both left and right foot, they were combined together by simple averaging. As a consequence, totally 11 studied group were formed, respectively noted as “A-W”, “A-J”, “A-WJ”, “A-W-I”, “A-W-C”, “A-J-I”, “A-J-C”, “A-W-Intra”, “A-W-Inter”, “A-J-Intra” and “A-J-Inter”, together with corresponding 11 control groups, noted as “H-W”, “H-J”, “H-WJ”, “H-W-I”, “H-W-C”, “H-J-I”, “H-J-C”, “H-W-Intra”, “H-W-Inter”, “H-J-Intra” and “H-J-Inter”. For example, the group labelled with “A-W” referred that the observation were focused on the ACL rupture patients during Walking, its similarity score was obtained by simply averaging the scores of both “AL-W” and “AR-W” in Experiment I; the group labelled with “A-J-C” was right the “AJ-C” in Experiment II, referred to the observation focused on the Contralateral side of ACL rupture patients while Jogging; the group labelled with “A-W-Intra” referred to the observation focused on the same ACL rupture subject during Walking, its similarity value was obtained by simply averaging the scores of both “AWL-Intra” and “AWR-Intra” in Experiment III; the group labelled with “A-J-Inter” referred to the observation focused on the intersubjects of ACL deficiency during Jogging, its similarity score was obtained by simply averaging the scores of both “JL-inter-A” and “JR-inter-A” in Experiment IV.

To detect whether the difference among studied conditions was significant or not, the Analysis of Variance (ANOVA) test was employed. All statistical tests were performed using SPSS 15.0 (SPSS Inc., Chicago, USA). The level of statistical significance was set at 0.05.

## Results

### Experiment I: On different motion patterns

**Fig 2:**
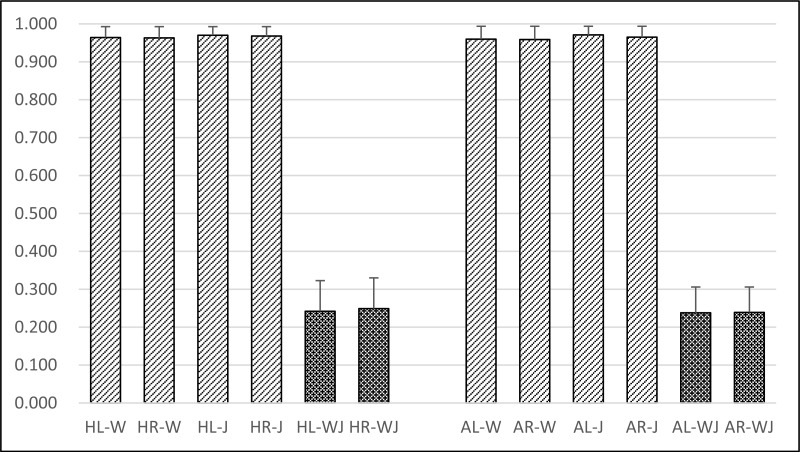
Investigation on human COP movements under the influences derived from motion pattern (walking and jogging).

Fig 2 shows the similarity scores of eight control groups and four target groups. Similarity scores of both the left and right side were 0.964 ± 0.029 (HL-W) and 0.963 ± 0.030 (HR-W) during walking, 0.970 ± 0.023 (HL-J) and 0.968 ± 0.025 (HR-J) during jogging for the healthy volunteers, respectively. For the ACL rupture patients they were 0.960 ± 0.034 (AL-W), 0.959 ± 0.035 (AR-W) during walking, and 0.971 ± 0.023 (AL-J), 0.965 ± 0.029 (AR-J) during jogging, respectively. However, for four target groups, the similarity scores between walking and jogging on the left and right sides were respectively 0.242 ± 0.081 and 0.249 ± 0.081 for the healthy volunteers, 0.238 ± 0.068 and 0.239 ± 0.067 for the ACL rupture patients, respectively.

“HL-W” referred to that the observation focused on *Left* side of *Healthy* subjects during *Walking*. “HR-W” referred to that the observation focused on *Right* side of *Healthy* subjects during *Walking*. “HL-J” referred to that the observation focused on *Left* side of *Healthy* subjects during *Jogging*. “HR-J” referred to that the observation focused on *Right* side of *Healthy* subjects during *Jogging*. “HL-WJ” referred that the observation were focused on *Left* side of *Healthy* subjects by calculating the similarity of COP trajectories between *Walking* and *Jogging*. “HR-WJ” referred that the observation were focused on *Right* side of *Healthy* subjects by calculating the similarity of COP trajectories between *Walking* and *Jogging*.

### Experiment II: On ipsilateral and contralateral

**Fig 3:**
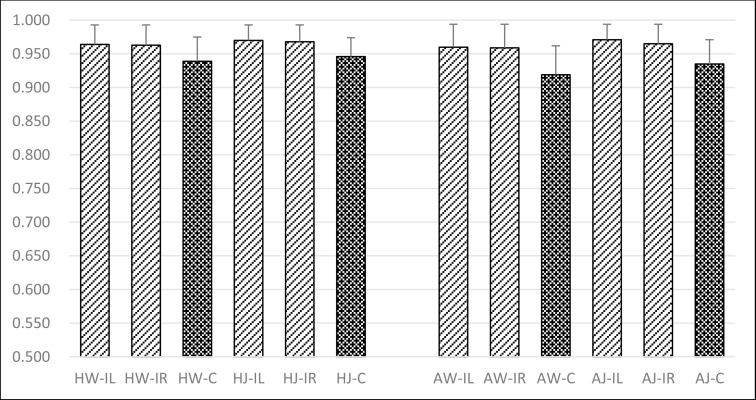
Comparison on the characteristics of human COP movements between ipsilateral sides and contralateral sides.

Fig 3 showed the similarity scores of eight control groups and four target groups. Similarity scores of both the left and right side were 0.964 ± 0.029 (HW-IL) and 0.963 ± 0.030 (HW-IR) during walking, 0.970 ± 0.023 (HJ-IL) and 0.968 ± 0.025 (HJ-IR) during jogging for the healthy volunteers, respectively. For the ACL rupture patients they were 0.960 ± 0.034 (AW-IL), 0.959 ± 0.035 (AW-IR) during walking, and 0.971 ± 0.023 (AJ-IL), 0.965 ± 0.029 (AJ-IR) during jogging, respectively. However, for four target groups, the similarity scores between the contralateral sides during walking or jogging were 0.939 ±0.036 (HW-C) and 0.946 ± 0.028 (HJ-C) for the healthy volunteers, 0.919 ± 0.043 (AW-C) and 0.935 ± 0.036 (AJ-C) for the ACL rupture patients, respectively. The Analysis of Variance (ANOVA) test showed that the COP similarity scores between the contralateral sides were significantly reduced in comparison with those between ipsilateral sides during walking or jogging in both normal and ACL deficient subjects (p<0.01).

“HW-IL” referred to the observation focused on the Ipsilateral Left side of Healthy subjects during Walking. “HW-IR” referred to the observation focused on the Ipsilateral Right side of Healthy subjects during Walking. “HW-C” referred to the observation that focused on the Contralateral sides of Healthy subjects by calculating the similarity of COP trajectories during Walking. “HJ-IL” referred to the observation focused on the Ipsilateral Left side of Healthy subjects during Jogging. “HJ-IR” referred to the observation focused on the Ipsilateral Right side of Healthy subjects during Jogging. “HJ-C” referred to the observation that focused on the Contralateral sides of Healthy subjects by calculating the similarity of COP trajectories during Jogging.

### Experiment III: On intrasubject and intersubject

**Fig 4:**
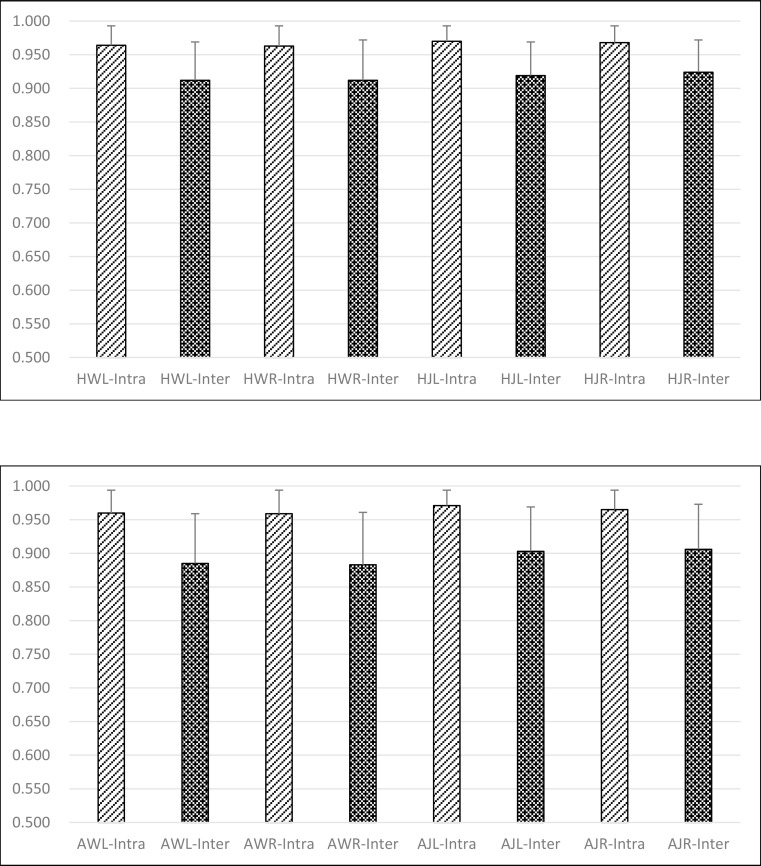
Comparison on the differences of human COP movements between intrasubject and intersubject. (a)For healthy volunteers; (b) For ACL-ruptured patients.

Fig 4 showed the similarity scores between the same subjects in the healthy group were 0.964 ± 0.029 (HWL-Intra) and 0.963 ± 0.030 (HWR-Intra) during walking, and 0.970 ± 0.023 (HJL-Intra) and 0.968 ± 0.025 (HJR-Intra) during jogging, respectively. The similarity scores between different subjects in the healthy group were 0.912 ± 0.057 (HWL-Inter) and 0.912 ± 0.060 (HWR-Inter) during walking, and 0.919 ± 0.050 (HJL-Inter) and 0.924 ± 0.048 (HJR-Inter) during jogging, respectively. For the same subjects of the ACL rupture patients, the similarity scores were 0.960 ± 0.034 (AWL-Intra) and 0.959 ± 0.035 (AWR-Intra) during walking, and 0.971 ± 0.023 (AJL-Intra) and 0.965 ± 0.029 (AJR-Intra) during jogging, respectively. Between different subjects of the ACL rupture patients, they were 0.885 ± 0.074 (AWL-Inter) and 0.883 ± 0.078 (AWR-Inter) during walking, 0.903 ± 0.066 (AJL-Inter) and 0.906 ± 0.067 (AJR-Inter) during jogging, respectively. The Analysis of Variance (ANOVA) test showed that the COP similarity scores between different subjects were significantly reduced in comparison to those of the same subjects during walking or jogging in both normal and ACL deficient subjects (p<0.001).

“HWL-Intra” referred to the observation focused on the Left side of the same Healthy subjects during Walking. “HWL-Inter” referred to the observation focused on the Left side of the Healthy Intersubjects by calculating the similarity of COP trajectories during Walking. “HWR-Intra” referred to the observation focused on the Right side of the same Healthy subjects during Walking.“HWR-Inter” referred to the observation focused on the Right side of the Healthy Intersubjects by calculating the similarity of COP trajectories during Walking.

“HJL-Intra” referred to the observation focused on the Left side of the same Healthy subjects during Jogging. “HJL-Inter” referred to the observation focused on the Left side of the Healthy Intersubjects by calculating the similarity of COP trajectories during Jogging. “HJR-Intra” referred to the observation focused on the Right side of the same Healthy subjects during Jogging.“HJR-Inter” referred to the observation focused on the Right side of the Healthy Intersubjects by calculating the similarity of COP trajectories during Jogging.

### Experiment IV: The influences derived from ACL deficiency

**Fig 5:**
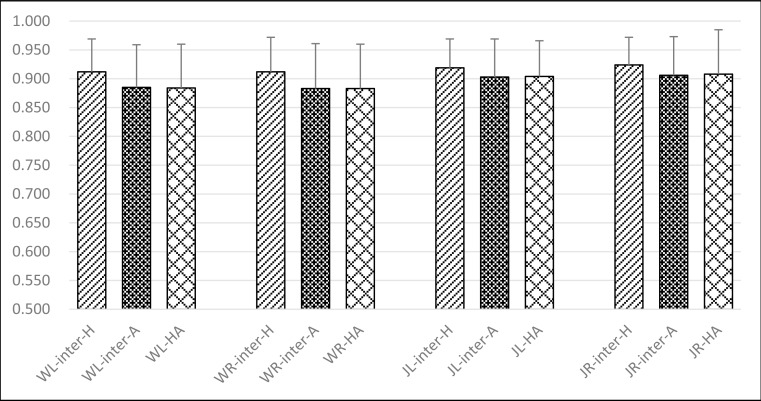
Illustration on the influences derived from ACL deficiency by comparing with the COP movements of healthy volunteers.

Fig 5 showed the similarity scores for both left side and right side of the healthy volunteers were 0.912 ± 0.057 (WL-inter-H) and 0.912 ± 0.060 (WR-inter-H) during walking, 0.919 ± 0.050 (JL-inter-H) and 0.924 ± 0.048 (JR-inter-H) during jogging in the healthy group, respectively. For the ACL rupture patients they were 0.885 ± 0.074 (WL-inter-A) and 0.883 ± 0.078 (WR-inter-A) during walking, 0.903 ± 0.066 (JL-inter-A) and 0.906 ± 0.067 (JR-inter-A) during jogging, respectively. For the comparison between the healthy volunteers and ACL rupture patients, they were 0.884 ± 0.076 (WL-HA) and 0.883 ± 0.077 (WR-HA) during walking, 0.904 ± 0.062 (JL-HA) and 0.908 ± 0.077 (JR-HA) during jogging, respectively. The Analysis of Variance (ANOVA) test showed that the COP similarity scores of the healthy volunteers were significantly higher than those of the ACL rupture patients during walking or jogging (p<0.001). The similarity scores between ACL rupture patients and healthy subjects were also significantly reduced compared to those between healthy subjects.

“WL-inter-H” referred to the observation focused on the Left side of the Healthy subjects by calculating the similarity of COP trajectories during Walking. “WL-inter-A” referred to the observation focused on the Left side of the ACL-ruptured patients by calculating the similarity of COP trajectories during Walking. “WL-HA” referred to the observation focused on the Left side between Healthy volunteers and ACL-ruptured patients by calculating the similarity scores of COP trajectories during Walking.

“WR-inter-H” referred to the observation focused on the Right side of the Healthy subjects by calculating the similarity of COP trajectories during Walking. “WR-inter-A” referred to the observation focused on the Right side of the ACL-ruptured patients by calculating the similarity of COP trajectories during Walking. “WR-HA” referred to the observation focused on the Right side between Healthy volunteers and ACL-ruptured patients by calculating the similarity scores of COP trajectories during Walking.

“JL-inter-H” referred to the observation focused on the Left side of the Healthy subjects by calculating the similarity of COP trajectories during Jogging. “JL-inter-A” referred to the observation focused on the Left side of the ACL-ruptured patients by calculating the similarity of COP trajectories during Jogging. “JL-HA” referred to the observation focused on the Left side between Healthy volunteers and ACL-ruptured patients by calculating the similarity scores of COP trajectories during Jogging. “JR-inter-H” referred to the observation focused on the Right side of the Healthy subjects by calculating the similarity of COP trajectories during Jogging. “JR-inter-A” referred to the observation focused on the Right side of the ACL-ruptured patients by calculating the similarity of COP trajectories during Jogging. “JR-HA” referred to the observation focused on the Right side between Healthy volunteers and ACL-ruptured patients by calculating the similarity scores of COP trajectories during Jogging.

**Fig 6:**
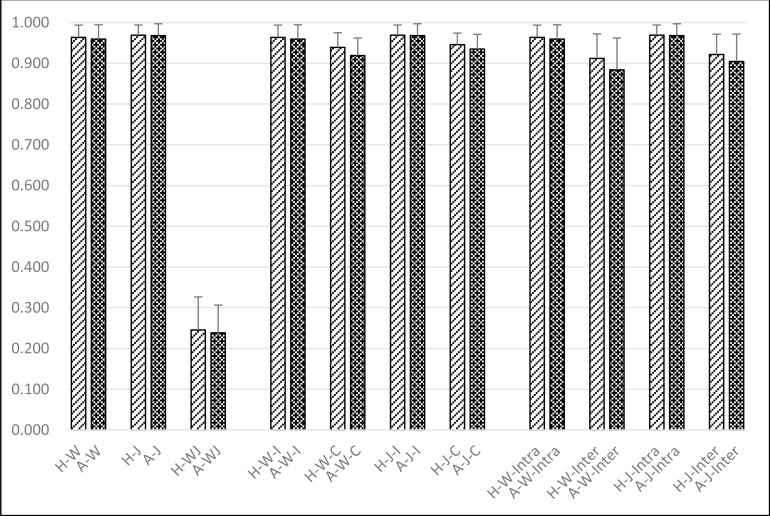
Re-exhibition the influences derived from ACL deficiency comparing with healthy volunteers under totally 11 conditions.

From the re-exhibition of these results (in Fig 6), it can be seen that all studied groups of ACL-ruptured patients had significantly lower similarity scores comparing to that of the corresponding control groups in a completely consistent manner.

“H-W” referred that the observation were focused on the Healthy subjects during Walking, its similarity score was obtained by simply averaging the scores of both “HL-W” and “HR-W” in Experiment I; “H-J-C” referred to the observation focused on the Contralateral side of the Healthy subjects while Jogging; “H-W-Intra” referred to the observation focused on the same the Healthy subjects during Walking; “H-J-Inter” referred to the observation focused on the intersubjects of the Healthy subjects during Jogging.

“A-W” referred that the observation were focused on the ACL rupture patients during Walking, its similarity score was obtained by simply averaging the scores of both “AL-W” and “AR-W” in Experiment I; the group labelled with “A-J-C” is right the “AJ-C” in Experiment II, referred to the observation focused on the Contralateral side of ACL rupture patients while Jogging; the group labelled with “A-W-Intra” referred to the observation focused on the same ACL rupture subject during Walking, its similarity value was obtained by simply averaging the scores of both “AWL-Intra” and “AWR-Intra” in Experiment III; the group labelled with “A-J-Inter” referred to the observation focused on the intersubjects of ACL deficiency during Jogging, its similarity score was obtained by simply averaging the scores of both “JL-inter-A” and “JR-inter-A” in Experiment IV.

## Discussion

The purpose of this study was to examine the similarity of the COP and its change during walking and jogging following the ACL deficiency. In accordance with our hypotheses, COP similarity can distinguish walking from jogging. In addition, the COP similarity would be changed in ACL-ruptured patients compared with healthy volunteers. These findings provide a reliable dataset for COP characteristics during walking and jogging in healthy volunteers and ACL-ruptured patients.

Walking and jogging are the most common activities that humans perform during activity of daily living, sports and rehabilitation. Given that an average adult takes more than 5,000 steps per day [16], even slight adaptations in ACL injured patients could have a significant cumulative effect on an ACL-deficient knee. The percentage of the stance phase varies depending on gait speed—about 60% with walking, 40% with running, There is a period of double limb support in which both feet are on the ground during walking, while a period of double float in which both feet are off the ground during jogging [9]. The COP measurements are clinically applicable since they are quick, non-invasive, and objective (see Fig 1). Understanding of the dynamic COP trajectory can provide additional information related to lower extremity moments and balance control during gait [17,18]. A symmetrical movement of the lower limbs characterizes a normal gait, and this symmetry can be reduced in pathological gait. The COP can be measured using pressure platforms, which has been used to investigate the medial–lateral stability of the foot [19] and effectiveness of foot orthoses [20]. However, similarity of the COP data was barely reported. Intra-subject variability and the effects of parameters such as walking/ jogging, healthy/ pathology require investigation before any safe conclusions can be drawn from the COP data. Little is known about the COP patterns in ACL deficiencies. One aim of the present study was to investigate COP similarity in a group of ACL deficient patients.

Similarity scores between walking and jogging COP trajectories were lower than 0.25 (see Fig 2) which indicated COP trajectories could distinguish walking from jogging. Moreover, similarity scores between the same sides were significantly higher than those between different sides (see Fig 3). During the same motion, similarity scores between the intersubjects were high and those between the intrasubjects were even higher (see Fig 4). These would be of importance in the future clinical research and application.

Although similarity scores between the COP trajectories in healthy volunteers were high (0.939±0.036 during walking, 0.946±0.028 during jogging), there was a significant difference between left and right sides not only during walking but also during jogging (p<0.0001). Our results showed that asymmetry and laterality (limb preference for a particular task) previously recognized not only in temporal and force parameters [21,22,23] but also occurs in the COP pattern (see Fig 3). The assumption for gait symmetry resides in the continuous nature of walking or jogging that each limb undergoes a swing and stance phase. However, the theory behind functional gait asymmetry was based on the idea that each leg was tuned to a specific gait task during the stance phase. Sadeghi et al[22] noted that one limb was more responsible for forward propulsion, whereas the other limb provided support and stability during normal walking. This conclusion was similar to prior research that noted the left limb was responsible for medio-lateral balance[21] and support [23] while the right leg provided more forward propulsion in young adults[24]. However, notably absent from these studies was the degree to which asymmetry exists in healthy or injured populations.

ACL deficiency would result in knee joint instability and increase internal rotation of the tibia during the gait cycle[4], which would lead to abnormal spatiotemporal parameters due to an automatic coupling between knee and foot [5,6]. In comparison with the healthy volunteers, ACL rupture patients showed that the COP similarity scores were significantly reduced during walking and jogging (p<0.001) (see Fig 5). We could conclude that there was a significant difference related to COP similarity between the ACL rupture patients and the normal subjects, which may lead to an asymmetrical gait. In addition, some compensation strategies occur during stance on the sound leg, which produces a modified COP pattern with respect to that of normal subjects. It is possible that an adaptation with the sound side and the injured side, which modifies its behavior to improve stability and performance of gait. From Experiment I, II, III, IV and the re-exhibition of these results illustrated in Fig 6, it could be seen that all studied groups of ACL-ruptured patients had significantly lower similarity scores comparing to that of the corresponding control groups in a completely consistent manner. Symmetry can be defined as an exact correspondence between opposite halves of a figure or form. Conversely, asymmetry was any deviation from this ideal structure. In accordance with previous studies [25], we demonstrated that the ACL deficient patients seemed to use a compensation strategy to reduce similarity. This adaptation mechanism to reduce the challenge of the motor task was also observed in ACL deficient patients during level walking [25]. It was possible that the balance of pressures during the stance phase was maintained by the muscular control of foot movement and/or the contribution of proximal body segments to the pressure distribution. Measurements of muscle activity and more detailed kinematic analysis would be required to investigate these suggestions.

In summary, the present study showed that COP similarity from the dynamic pedobarography not only provides a practical and objective way to evaluate dynamic function of walking and jogging that was not studied exclusively before, but also helps in evaluating and guiding future rehabilitative and surgical techniques in ACL deficient patients. However, previous studies have established that ACL rupture induces variability in postural control and gait patterns. ACL deficiency adopts certain strategies to maintain dynamic stability and balance. The modulating mechanism for postural control is complex, possibly affecting the COP trajectory. Interpretations of these findings should not only generalize the effect of ACL rupture, but also consider the variation in dynamic postural control during motion. Further studies are needed to clarify the relationship between dynamic stability and ACL rupture. Furthermore, large-scale measurements of muscle activity and more detailed kinematic studies in both ACL-intact and deficient subjects are necessary to develop population reference values to improve our general clinical understanding of individual variations in the biomechanical coupling between foot and knee.

## Conclusion

This investigation showed the COP similarity during walking was significantly different from that during jogging. ACL deficiency tends to display a more variable COP curve compared to healthy adults. These findings regarding the COP similarity for ACL rupture provided more information for dynamic foot function and gait performance, potentially assisting in clinical intervention assessment for the ACL rupture.

## Supporting Information

S1 Dataset The detail information of plantar pressure data of some subjects included for analysis. (ZIP)
